# Paclitaxel Induces Apoptosis in Breast Cancer Cells through Different Calcium—Regulating Mechanisms Depending on External Calcium Conditions

**DOI:** 10.3390/ijms15022672

**Published:** 2014-02-17

**Authors:** Zhi Pan, Andrew Avila, Lauren Gollahon

**Affiliations:** 1 Department of Biological Sciences, Texas Tech University, Lubbock, TX 79409, USA; E-Mails: zhi_pan@yahoo.com (Z.P.); xydane@gmail.com (A.A.); 2 Texas Tech University Imaging Center, Texas Tech University, Lubbock, TX 79409, USA

**Keywords:** breast cancer, apoptosis, paclitaxel, external calcium, endoplasmic reticulum

## Abstract

Previously, we reported that endoplasmic reticulum calcium stores were a direct target for paclitaxel initiation of apoptosis. Furthermore, the actions of paclitaxel attenuated Bcl-2 resistance to apoptosis through endoplasmic reticulum-mediated calcium release. To better understand the calcium-regulated mechanisms of paclitaxel-induced apoptosis in breast cancer cells, we investigated the role of extracellular calcium, specifically; whether influx of extracellular calcium contributed to and/or was necessary for paclitaxel-induced apoptosis. Our results demonstrated that paclitaxel induced extracellular calcium influx. This mobilization of extracellular calcium contributed to subsequent cytosolic calcium elevation differently, depending on dosage. Under normal extracellular calcium conditions, high dose paclitaxel induced apoptosis-promoting calcium influx, which did not occur in calcium-free conditions. In the absence of extracellular calcium an “Enhanced Calcium Efflux” mechanism in which high dose paclitaxel stimulated calcium efflux immediately, leading to dramatic cytosolic calcium decrease, was observed. In the absence of extracellular calcium, high dose paclitaxel’s stimulatory effects on capacitative calcium entry and apoptosis could not be completely restored. Thus, normal extracellular calcium concentrations are critical for high dose paclitaxel-induced apoptosis. In contrast, low dose paclitaxel mirrored controls, indicating that it occurs independent of extracellular calcium. Thus, extracellular calcium conditions only affect efficacy of high dose paclitaxel-induced apoptosis.

## 1. Introduction

Calcium, an important cellular regulator, has a dual role in the fate of the cell. While stable calcium homeostasis is necessary for normal cell survival, impaired calcium homeostasis is toxic to cells and may induce cell death [1–6]. Among various forms of cell death, apoptosis is a major mechanism utilized by chemotherapy agents to kill cancer cells [7–9]. Therefore, the calcium-apoptosis link provides a key potential target for developing and optimizing cancer therapies [10–21]. Our previous work showed that paclitaxel, an important chemotherapy agent for breast cancer, can directly attack the internal endoplasmic reticulum (ER) calcium store to release apoptosis—promoting calcium signals depending on dosages [22]. In this study, we focus on the role of external calcium to fully understand the calcium—regulated mechanisms of paclitaxel-induced apoptosis.

Previous studies [23–25] in paclitaxel-induced apoptosis mainly focused on the mitochondrial apoptotic pathways. Paclitaxel, directly or indirectly, releases apoptogenic factors such as Cyto C into the cytosol through mitochondrial membrane permeabilization (MMP) or mitochondrial permeability transition pore (MPTP) openings. Cyto C can activate caspases, resulting in apoptosis. However, how paclitaxel induces MMP or MPTP remains unclear. Calcium, one of primary triggers to induce MMP or MPTP, provides a possible explanation. Cell-free experiments [26] on isolated purified mitochondria showed that extremely high dose of paclitaxel (2 × 10^−5^ M) directly damages the outer and inner mitochondrial membranes and results in the release of apoptotic factors from the mitochondria, inducing the mitochondrial apoptotic pathway without an initial increase in mitochondrial matrix calcium levels ([Ca^2+^]_mito_) or cytosolic levels ([Ca^2+^]_c_). As a result of this process, calcium is also released from the mitochondria matrix into the cytosol. The increased cytosolic calcium level in turn, has a positive influence on apoptosis. Using a neuron cell culture system, Mirononv *et al.* [27], showed this mitochondrial calcium release is probably promoted by the stabilization of paclitaxel to the microtubules associated with the outer mitochondrial membrane, although the mechanism is unclear. However, these studies did not address the effects of commonly used doses (10^−7^~10^−6^ M) on breast cancer cell systems.

The ER, an important upstream gateway, is more important in calcium apoptosis handling than mitochondria. However, the mechanism by which paclitaxel engages the ER apoptotic pathway is still unclear. Previous studies [25,28] showed that paclitaxel treatment results in the production of intermediates such as lipid ceramide through *de novo* pathways from the lipid membranes and reactive oxygen species (ROS) from the mitochondria matrix. Recently, ceramide and ROS were reported to trigger a massive calcium release from the ER, initiating a downstream mitochondrial apoptotic pathway in a strictly ER calcium-dependent manner [13,14,29]. More recently, Baggott *et al.* [30] demonstrated that if PMCA2/calcineurin interaction is disrupted, the calcineurin/NFAT pathway is activated, resulting in the upregulation of the pro-apoptotic protein Fas Ligand and loss of cell viability due to an increase in cell apoptosis. This PMCA2/calcineurin disruption augmented paclitaxel-mediated cytotoxicity of breast tumor cells. Taken together these studies suggested that paclitaxel may engage the ER calcium pathway indirectly. Our previous study showed a direct relationship involving paclitaxel-induced apoptosis and ER-calcium [22] based on dosage and duration of exposure. Furthermore, paclitaxel may act as an ER stress signal when it binds and inhibits Bcl-2 protein localized on the ER membrane [31,32]. The effect of paclitaxel on Bcl-2 as well as possible alterations in Bcl-2/calcium dynamics may represent another mechanism of how paclitaxel induces apoptosis-related calcium signals. Recently, we demonstrated the “Tug-of-War” that exists between paclitaxel and BCL2 for calcium regulation—determining whether apoptosis occurs [33]. In this study we investigated whether paclitaxel induces calcium influx from the extracellular medium, modulating apoptosis events.

Specifically, our study addressed two major questions. First, we were interested in determining whether calcium influx from the extracellular fluid (ECF) contributes to paclitaxel-induced apoptosis. The second question addressed was whether external calcium is necessary for paclitaxel to induce apoptosis. We hypothesized that external calcium influx contributes to paclitaxel-induced apoptosis through coupling to paclitaxel-induced ER calcium release based on the following reasons. As shown in our previous study, ER calcium release, upon stimulation, results in the depletion of the ER calcium store. This can increase external calcium influx through channels and/or exchangers on the plasma membrane [34]. This increased external calcium influx coupled to ER calcium release is called capacitative calcium entry (CCE), which serves to generate a sustained cytosolic calcium elevation and to refill the depleted ER calcium stores [11,18]. As a result, CCE provides a potential mechanism for involvement of external calcium in apoptosis—promoting cytosolic calcium changes upon stimulus.

Furthermore, our previous study showed that paclitaxel can directly attack the ER store causing rapid release of ER calcium into the cytosol, resulting in a gradual ER calcium depletion (in hours), and consequently provide the potential trigger to increase calcium influx from the medium into the cytosol [22]. Therefore, paclitaxel treatment may also stimulate CCE coupled with ER calcium release to maintain the global cytosolic calcium elevation, which signals downstream activation of caspase or other calcium-dependent enzymes ultimately inducing or promoting apoptosis. Taken together, these data suggest that paclitaxel may mobilize external calcium through CCE to promote subsequent apoptosis.

To test the above hypothesis, we first investigated whether paclitaxel can modulate calcium influx from the medium into the cytosol through CCE. Next we assessed the contribution of the changed CCE to paclitaxel-induced apoptosis. After the paclitaxel-induced changes in CCE and their effects on the subsequent apoptosis were determined, we analyzed whether external calcium was necessary for paclitaxel-induced apoptosis. For the second part of this study, we measured paclitaxel-induced changes between internal ER calcium, external calcium and cytosolic calcium concentrations in the absence of external calcium to better understand the relationship of paclitaxel in calcium-dependent apoptosis.

In summary, using a cell-culture model, this research examined whether external calcium is a sufficient and/or necessary regulator in paclitaxel-induced apoptosis and the underlying calcium-regulating process to fully understand the mechanism of paclitaxel in breast cancer chemotherapy.

## 2. Results and Discussion

### 2.1. Effects of Paclitaxel on the CCE of the Cell Depends on Dosage

**Figure 1.**
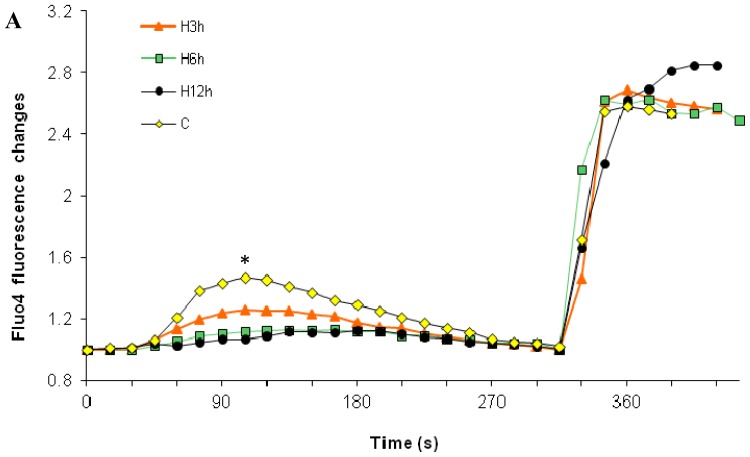

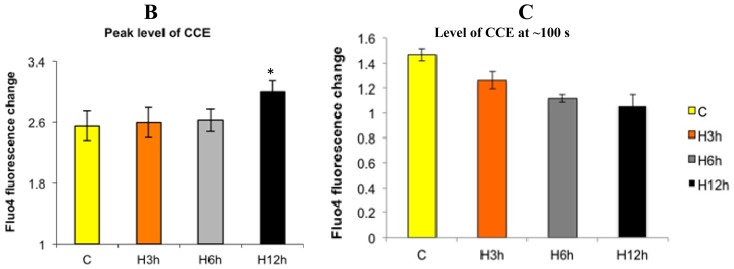
Effects of high dose paclitaxel on the cell’s ability to influx external Ca^2+^. Under normal calcium conditions, cells were analyzed after treatment of high dose paclitaxel at 10^−6^ M (3, 6, and 12 h). Arrows show the addition of 3 μM TG at 15 s and 2 mM CaCl_2_ at 300 s. (**A**) Time-response curve (mean) was determined from data generated in three independent tests; (**B**) From the top curve, peak levels of capacitative calcium entry (CCE), external Ca^2+^ influx, were represented by mean ± SD and statistical analysis was performed; (**C**) From the top curve, levels of CCE at 100 s, indicated by *.

As discussed in the introduction, the cellular calcium influx from the medium into the cytosol may be changed after paclitaxel treatment, and coupled to paclitaxel-induced gradual ER calcium depletion. To test the hypothesis, we first needed to differentiate effects of paclitaxel treatment on the released ER calcium and influx of external calcium. To accomplish this, the TG-CaCl_2_ method was applied. TG is a highly specific ER calcium pump (SERCA) inhibitor, which irreversibly blocks calcium entry into the ER. Results showed that the cell’s ability to influx calcium from the medium through CCE was not significantly increased until the 12 h treatment of high dose paclitaxel (Figure 1, 20% increase, *p* < 0.05). This CCE increase followed a paclitaxel-induced, gradual, ER calcium depletion, which first appeared after 3 h treatment (*p* < 0.05) and then became more significant after 6 and 12 h treatment (*p* < 0.01). Both paclitaxel-induced increase in external calcium influx coupled with the ER calcium depletion could serve as death signals, inducing apoptosis.

**Figure 2.**
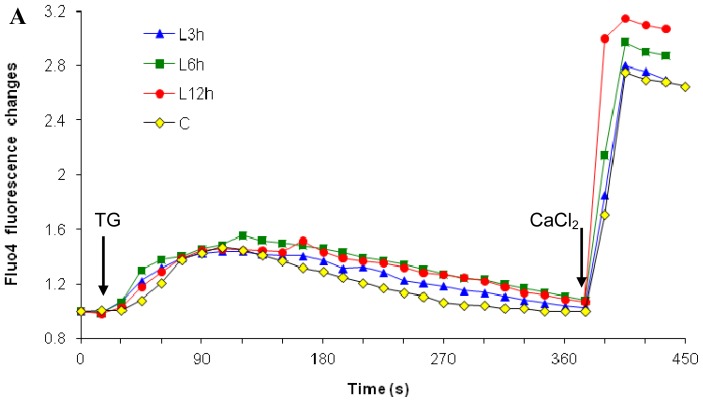

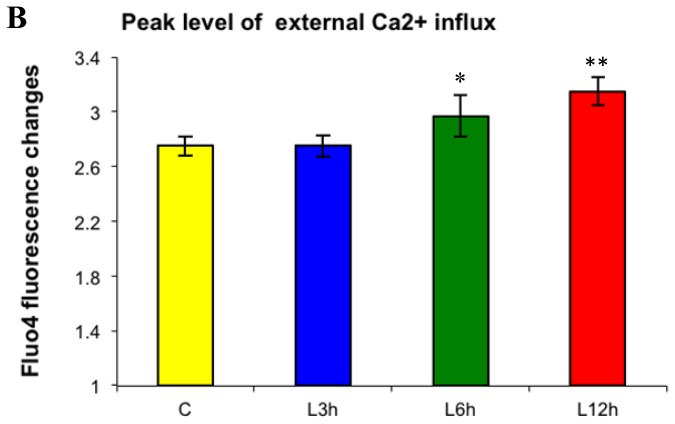
Effects of low dose paclitaxel on the cell’s ability to influx external Ca^2+^. Under normal calcium conditions, cells were analyzed after treatment of low dose paclitaxel at 10^−7^ M (3, 6, and 12 h). Arrows show the addition of 3 μM TG and 2 mM CaCl_2_. (**A**) Time-response curve (mean) was determined from data generated in three independent tests; (**B**) Peak levels of external Ca^2+^ influx for data from **A** were represented by mean ± SD, and statistical analysis was performed. * *p* < 0.05; **p < 0.01.

In contrast to high dose, the low dose paclitaxel at 10^−7^ M was observed to induce an increase in external calcium influx earlier, starting from 6 h treatment (a 15% increase, Figure 2) and reaching a more significant level after 12 h treatment (a 28% increase, *p* < 0.05, Figure 2). However, low dose paclitaxel did not induce a depletion of the ER calcium stores—at least at this time point (Figure 2). Therefore, low dose paclitaxel triggered mechanism independent of ER calcium release, causing external calcium influx. It is possible that the external calcium influx inducts or promotes subsequent apoptosis.

Our results showed that paclitaxel can enhance the cell’s influx of external calcium, and the contribution of enhanced calcium influx and underlying mechanisms depend on dosage of paclitaxel. High dose paclitaxel (10^−6^ M, Figure 1) first induced ER calcium release, which gradually resulted in ER calcium depletion. The ER calcium depletion then triggered the increase in CCE (external calcium influx). Therefore, high dose paclitaxel-enhanced CCE depends on the involvement of ER calcium release.

In contrast, although low dose paclitaxel (10^−7^ M, Figure 2) can also increase external calcium influx, it did not involve the ER calcium-regulated pathway, since low dose paclitaxel does not significantly affect ER calcium release. These findings demonstrated that paclitaxel can stimulate external calcium influx, but the mobilization of external calcium contributes to subsequent cytosolic calcium elevation differently, depending on dosage. The next step was to investigate whether external calcium is necessary for the cytosolic calcium elevation.

### 2.2. Effects of Removing External Calcium Influx on Subsequent Apoptosis Using Two Different Methods—The External Calcium Chelator BAPTA, and Calcium-Free Medium

**Figure 3.**
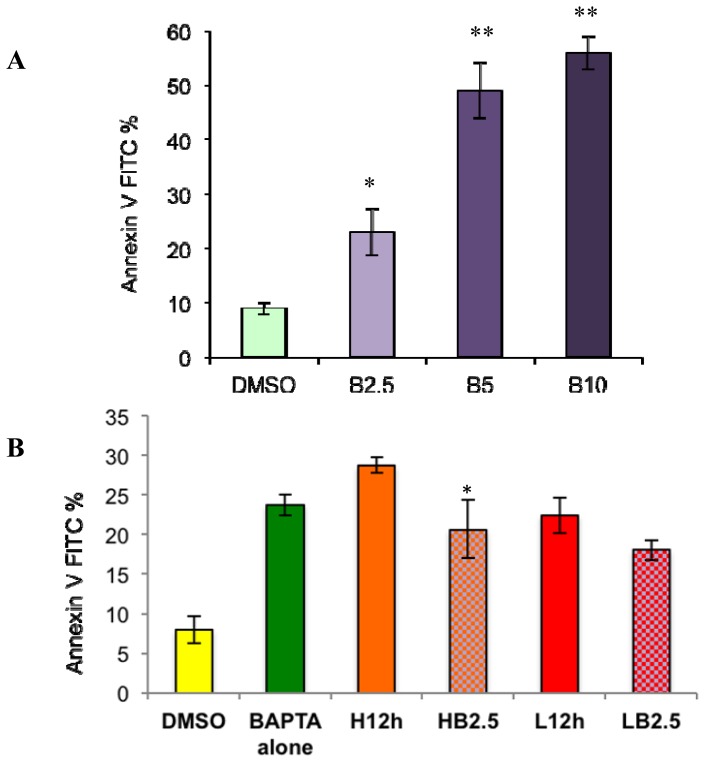
Effects of the external Ca^2+^ chelator BAPTA on paclitaxel-induced apoptosis. Total apoptosis level was measured by Annexin V-FITC assay. (**A**) is total apoptosis induced by BAPTA alone after 12 h exposure; (**B**) is total apoptosis level induced by high or low dose paclitaxel (H12h, L12h) with or without 2.5 mM BAPTA (HB2.5, LB2.5) determined after 12 h. The results (mean ± SD) were determined from data generated in three independent tests. One-way ANOVA plus Tukey’s honestly significant difference (HSD) test were performed for results of each panel. * indicates significance at *p* < 0.05 and ** indicates significance at *p* < 0.01.

To determine whether external calcium influx was required for paclitaxel-induced apoptosis, the action of the external calcium chelator, BAPTA, was studied at three different doses: 2.5, 5, 10 mM. BAPTA without acetoxymethyl (AM) esters cannot penetrate into cells. It specifically chelates extracellular free Ca^2+^ and thus inhibits the Ca^2+^ action. Therefore, the pretreatment of BAPTA was used to test whether paclitaxel-induced apoptosis was dependent on extracellular free calcium. BAPTA was dissolved into distilled water to make a 500 mM stock solution, which was filtered and then diluted into media to make three different final concentrations: 2.5, 5 and 10 mM. These dosages were chosen from relevant previous studies [35–39] (see Figure 3). Pretreatment with BAPTA alone for 1 h did not induce obvious apoptosis. However, in order to maintain cells in an external calcium free environment to evaluate its effect on paclitaxel-induced apoptosis, BAPTA treatment was continued in control medium for 12 h. Significant apoptosis was observed after 12 h BAPTA treatment (Figure 3), especially at 5 and 10 mM (*p* < 0.01). Fifty percent of the cells detached from the plate bottom in the presence of 5 and 10 mM BAPTA (data not shown). This suggests that survival and attachment of breast cancer cells depend on normal external calcium levels, similar to other cell types [40]. Another alternative explanation is that long-term BAPTA treatment may induce other potential mechanisms stimulating apoptosis, instead of external calcium removal.

**Figure 4.**
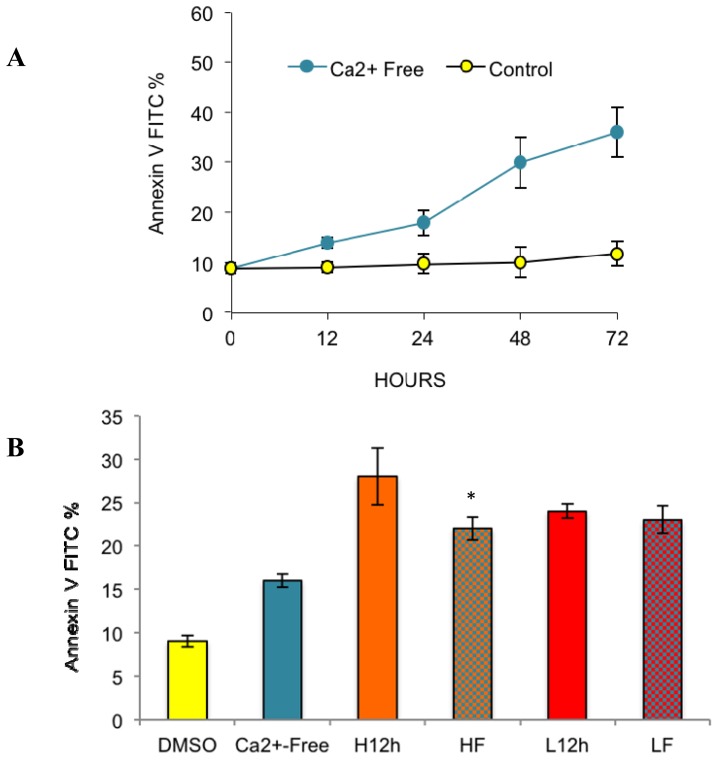
Effects of Ca^2+^ free medium on paclitaxel-induced apoptosis. Total apoptosis level was measured by Annexin V-FITC assay. (**A**) is total apoptosis induced by Ca^2+^ free medium alone; (**B**) is the total apoptosis level induced by high or low dose paclitaxel in control medium (H12h, L12h) or Ca^2+^ free medium (HF, LF). The results (mean ± SD) were determined from data generated in three independent tests. One-way ANOVA plus Tukey’s HSD test were performed for results of each panel. * = significance at *p* < 0.05.

To test the two above possibilities, we used a calcium-free medium instead of BAPTA^+^ for further investigation. The media were identical as described in the methods, except for +/− calcium. Our results showed that calcium-free medium induced a level of apoptosis similar to BAPTA^+^ at 2.5 mM from 12 h (Figure 4, *p* < 0.05). The result clarified two points. First, that normal external calcium conditions are required to maintain cell survival in breast cancer cells, and long-term absence of external calcium alone can result in apoptosis. Second, BAPTA long-term treatment at higher doses (5 and 10 mM), involved some other mechanism rather than external calcium removal to induce apoptosis.

Next, we wanted to elucidate the relationship between the two apoptosis-generating factors (e.g., paclitaxel treatment and external calcium removal). Although either alone can induce apoptosis, no additive or synergistic effects on apoptosis were observed when cells were treated with paclitaxel in the absence of extracellular calcium (either by BAPTA^+^ or calcium-free medium). Specifically, at the lower dose, paclitaxel induced similar levels of apoptosis in both control and calcium-free medium (Figures 3 and 4). At the higher dose, paclitaxel-induced apoptosis in calcium-free medium was even lower than results from conditions with any single factor (Figure 4, *p* < 0.05). The results indicated that an antagonizing effect exists between the two apoptosis-generating factors, high dose paclitaxel and external calcium removal.

#### 2.2.1. Paclitaxel Has a Different Calcium-Regulating Action in the Absence of External Calcium

**Figure 5.**
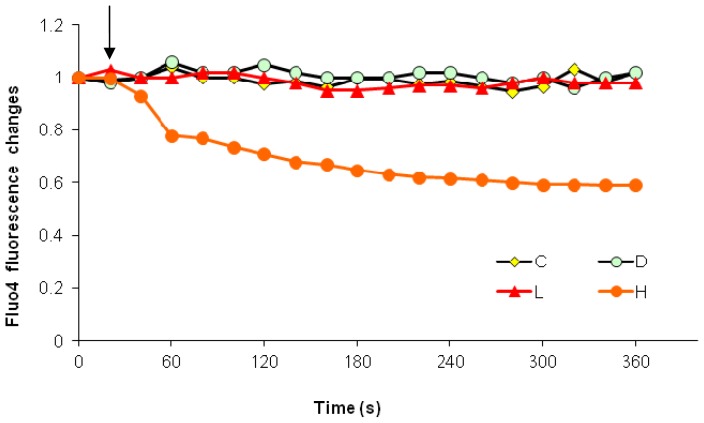
Paclitaxel-induced cytosolic Ca^2+^ decrease in Ca^2+^-free medium. The basal Fluo4 fluorescence level of all groups before treatment was similar and was expressed as 1. The **arrow** shows the time point at which agents (C—control, D—DMSO, L—Low dose paclitaxel, H—high dose paclitaxel) were added. The results (mean) were determined from data generated in three independent tests.

Under external calcium-free conditions, when high dose paclitaxel was added into a calcium-free medium, we observed a rapid, 40% decrease in the cytosolic calcium level (Figure 5). Low dose paclitaxel did not have a significant effect on cytosolic calcium level regardless of external calcium conditions.

**Figure 6.**
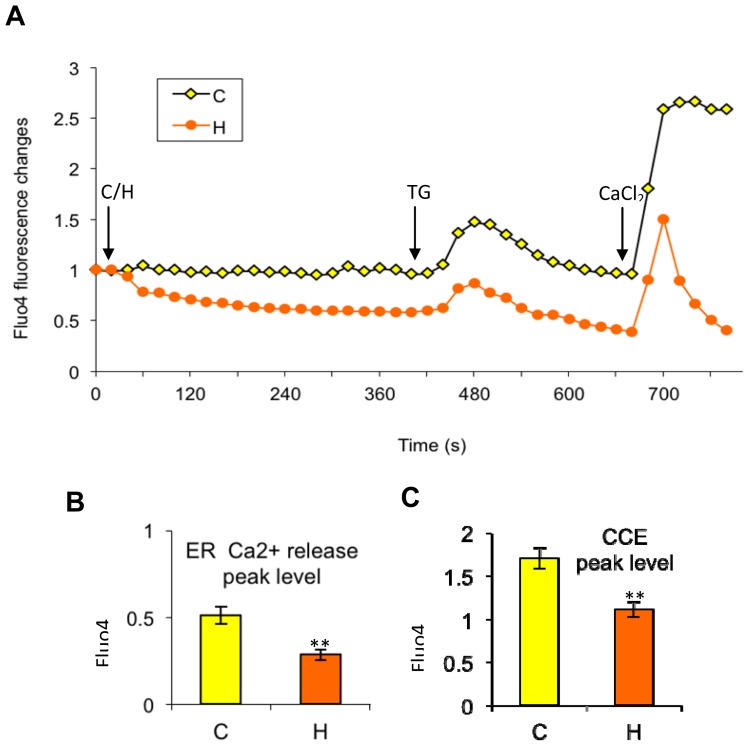
Effects of high dose paclitaxel (H) on the cytosolic Ca^2+^ levels in a Ca^2+^-free medium. (**A**) Time-response curves (mean) were determined from data generated in three independent tests. Before analyzing, cells were transferred to a Ca^2+^-free medium. Then high dose paclitaxel at 10^−6^ M was added (H) or not (C) into the medium, indicated by the first arrow. After waiting until fluo-4 levels stabilized (~5 min), TG (3 μM) and CaCl_2_ (2 mM) were added respectively, indicated by subsequent, labeled arrows. For the curves; (**B**) peak levels of endoplasmic reticulum (ER) Ca^2+^ release and (**C**) subsequent CCE were compared for control and high dose paclitaxel treated groups, which were represented by mean ± SD. ** indicates significance at *p* < 0.01.

Furthermore, we tackled the key question concerning where the cytosolic calcium goes during high dose paclitaxel treatment. Does it move into the internal ER stores or into the medium? Through a step-wise experimental progression (Figure 6), Enhanced Calcium Efflux or ECE was shown to be the most plausible explanation based on the following rationale: (1) The amplitude of cytosolic calcium increase induced by TG stimulation in the paclitaxel-treated group was only 60% of the control group, indicating that ER release was probably overcome by a cytosolic calcium extrusion mechanism; (2) the cytosolic calcium levels continued to decrease below the basal level after TG stimulation. This result excludes the possibility that calcium was pumped back to the ER because TG irreversibly blocked the SERCA pump; (3) the subsequent external calcium replenishment in the high dose paclitaxel-treated group resulted in a sharp cytosolic calcium increase only, followed by a sharp decrease, dropping back to unfilled levels without any sustained plateau. This is consistent with the observations that refilled calcium was removed from the cytosol very rapidly. Considering the kinetics of the mitochondrial uniporter, which takes up calcium from the cytosol normally, it may have been functionally overloaded by such large amounts of calcium entering the cytosol so quickly.

Taken together, these results suggest that the most likely mechanism of high dose paclitaxel in the absence of external calcium is an “Enhanced Calcium Efflux” model in which high dose paclitaxel stimulates calcium efflux immediately and thus leads to a cytosolic calcium decrease. This ECE can even inhibit TG-induced cytosolic increases through ER calcium release and subsequent CCE. This model is further supported by the data generated from our apoptosis measurements in the BAPTA^+^ and calcium-free medium.

In summary, in control medium with the normal external calcium conditions, paclitaxel caused an increase in external calcium influx to the cytosol, which promoted subsequent apoptosis. However, in the absence of external calcium, paclitaxel cannot mobilize the external calcium source, and the subsequent calcium-dependent apoptosis is inhibited. Since high dose paclitaxel and external calcium removal antagonize each other in the induction of apoptosis, we further dissected the relationship between them by testing whether external calcium removal directly mediated a high dose paclitaxel-induced cytosolic calcium change, which is the intermediate step in paclitaxel-induced apoptosis.

### 2.3. Effects of the Absence of External Calcium on Paclitaxel-Induced Cytosolic Calcium Responses

In our previous study, high dose paclitaxel induced a rapid cytosolic calcium elevation under normal external calcium conditions, whereas low dose paclitaxel did not [22]. As expected, this paclitaxel-induced cytosolic calcium elevation was not observed in the absence of external calcium. Furthermore, high dose paclitaxel resulted in a 40% decrease in cytosolic calcium levels (Figure 5, *p* < 0.001). This observation confirmed that high dose paclitaxel-induced cytosolic calcium increase is dependent upon external calcium. In addition, the rapid, dramatic decrease in cytosolic calcium suggests that cytosolic calcium flows to some other compartments. Considering the amplitude of the decrease, the ECF and ER calcium store are the most likely targets. Therefore, the next step was to investigate whether the cytosolic calcium flows back to the ER or flows outward to the ECF (in this experiment, the medium).

If paclitaxel returns cytosolic calcium back to the ER, then releasable calcium should accumulate in the ER, and this phenomenon should be reflected by the increase in ER calcium release, tested by TG. However, the amplitude of TG-induced ER calcium release was much lower in high dose paclitaxel treated cells, at only 60% of the control cells, thus excluding the ER calcium return (Figure 6, *p* < 0.01) explanation. This result is also consistent with the fact that paclitaxel (10^−6^ M) can directly induce the ER to release its stored calcium, thus lowering the available, releasable calcium.

An alternative explanation, Enhanced Calcium Efflux (ECE), was confirmed by the following. Figure 6 shows that after the ER was depleted, cytosolic calcium levels in the paclitaxel-treated group returned to the non-stimulated levels faster than the control group. Furthermore, cytosolic calcium levels continued to decrease even below the non-stimulated level (*p* < 0.05). At that time, the ER was depleted, TG inhibited ER uptake, and there was no refilling by external calcium. Therefore, the most plausible explanation for the continued rapid decrease was an enhanced calcium efflux.

The ECE mechanism was further supported after comparing the results of the external calcium replenishment between the paclitaxel-treated and control groups. The external calcium refilling only induced a 70% cytosolic calcium elevation in high dose paclitaxel-treated cells compared to the control (Figure 6, *p* < 0.01). A sharp decrease going back to unfilled levels without any sustained plateau followed, indicating that calcium efflux even overcame CCE. In addition, low dose paclitaxel and 0.5% DMSO did not show an obvious effect in the calcium-free condition (data not shown). This Enhanced Calcium Efflux, induced by high dose paclitaxel, explains the antagonism between high dose paclitaxel and external calcium removal on apoptosis and suggests that external calcium is necessary for paclitaxel-induced cytosolic calcium elevation. The possible causes and effects will be discussed in more detail later.

### 2.4. Paclitaxel’s Different Calcium-Regulating Actions Affect Its Efficacy to Induce Apoptosis

Our final objective was to test whether external calcium affects paclitaxel-induced apoptosis. Our findings indicate that paclitaxel has different efficacies in the absence and presence of external calcium. Although the removal of external calcium alone can induce high levels of apoptosis, high dose paclitaxel in calcium-free medium or BAPTA^+^ medium does not induce levels of apoptosis higher than controls. Instead, removal of external calcium from the medium either using BAPTA^+^ or calcium-free medium can even inhibit paclitaxel-induced apoptosis (Figures 3 and 4). The results support the contribution of external calcium in paclitaxel-induced apoptosis. Furthermore, our results show an ECE induced by high-dose paclitaxel, under external calcium-free conditions, inhibiting the cell’s ability to utilize external calcium for inducing apoptosis (Figures 4–6). Thus, high dose paclitaxel-induced apoptosis-promoting calcium influx, under normal external calcium conditions, cannot occur in calcium-free medium, further inhibiting paclitaxel’s efficacy to induce apoptosis.

Since high dose paclitaxel’s stimulatory effects on CCE cannot be restored even when external calcium has been replenished after paclitaxel treatment, normal extracellular calcium conditions first needs to be maintained in the medium to maximize the efficacy of high dose paclitaxel. In contrast, low dose paclitaxel induces similar level of apoptosis in control and calcium free medium, indicating that low dose paclitaxel-induced apoptosis is independent of external calcium conditions. The above findings reveal that the external calcium conditions can affect efficacy of high dose paclitaxel-induced apoptosis, but not low dose paclitaxel-induced apoptosis.

### 2.5. Alternative Explanations for Paclitaxel’s Different Calcium-Regulating Actions

How can paclitaxel stimulate calcium efflux so rapidly and greatly in calcium-free medium? Although there is an outward calcium concentration gradient across the plasma membrane in the absence of external calcium, it could not drive such a fast and significant calcium efflux considering that the cytosolic calcium level is already very low at resting levels. To address this question, we performed a computational analysis to identify potential docking partners of paclitaxel that might steer us towards a likely partner to help explain our results. This program was named *Artemis*. A detailed description of *Artemis*’ parameters is listed in the Experimental Section 3.7. The source code can be found in the Supplementary Material. *Artemis* was designed to save every docked configuration for paclitaxel. Thus, due to space constraints only the top 20 docking results are listed in Table 1. Interested readers can use the source code to recapitulate the results in order to determine if their molecule of interest interacts directly with paclitaxel.

The results generated from this docking program were very interesting. Of the top twenty molecules identified from this query, three human proteins were predicted to be highly probable direct paclitaxel-protein interactions. Specifically, take note of priority docking result #18. Using this data as a starting point, a plausible mechanism involving paclitaxel-induced apoptosis, ECE and p38 is proposed based on prior studies demonstrating that p38 is required for paclitaxel-induced apoptosis [41–43], as well as being implicated with calcium release [44,45]. The primary mechanism for calcium release is through the IP3R [46]. If this is disrupted, then ER stress response is attenuated and apoptosis may not be induced [47,48]. Additionally, studies have shown that modal switching is a major mechanism for regulating IP3R [49,50]. This modal switching may coincide with enhanced calcium entry in relation to paclitaxel dosage. Furthermore, interactions between p38 and paclitaxel at high doses may modulate CCE through the actions of CHOP [51], which downregulates BCL2 [52] and induces BIM [53,54]. In our previous study [33], we demonstrate that the ER calcium store is a common target for both paclitaxel and BCL2 protein. Paclitaxel directly associates with the endoplasmic reticulum to stimulate the release of calcium into the cytosol, contributing to the induction of apoptosis. However, BCL2 expression suppresses the cell’s pro-apoptotic response of endoplasmic reticulum calcium release, thus inhibiting susceptibility of cancer cells to undergo apoptosis. Depending upon dosage, a paclitaxel-induced stimulatory effect can overcome the BCL2-mediated inhibitory effect on endoplasmic reticulum calcium release, thus attenuating the resistance of BCL-2 to apoptosis. These interactions may have consequences for extracellular calcium efflux as a response to inhibition of calcium release from the ER [55,56]. For review of the interactions of these proteins with respect to ER, apoptosis, calcium, please see [57]. For review of mechanisms of calcium entry, please see [58]. This study provides new insight into calcium regulation in non-excitable cells [59–62]. Considering that the mammary epithelial cells secrete high amounts calcium during lactation [61], study of the calcium efflux mechanism needs more attention.

Another possible explanation may be the role of calcium efflux transporters. Although outside the scope of the present study, their potential involvement cannot be ignored. Normally, calcium efflux is used to maintain low cytosolic calcium levels against high external calcium levels through two major types of calcium transporters on the plasma membrane, the plasma membrane calcium pump (PMCA) and sodium-calcium exchanger (NCX) [11,18]. In non-excitable cells such as breast epithelial cells, NCX is the major transporter for calcium efflux, although its stimulatory efflux in the absence of external calcium has not been reported. Furthermore, NCX kinetics are more rapid in transporting calcium across the plasma membrane [63]. Hence, NCX is also a probable mechanism of paclitaxel-induced calcium efflux in the absence of external calcium and needs further study.

## 3. Experimental Section

### 3.1. Cell Culture and Reagents

The breast cancer cell line MDA-MB-468 (M468), obtained from the American Tissue Culture Center (ATCC, Manassas, VA, USA), was analyzed in this study. DMEM medium with normal external calcium levels (1.8 mM) was the “control medium” used to compare against the calcium free medium (Sigma, St. Louis, MO, USA). All of other culture conditions were similar to those described in our previous study [22].

M468 cells were chosen for two reasons. First, it is negative for endogenous the anti-apoptotic protein Bcl-2, and thus the effects of Bcl-2 on calcium homeostasis were eliminated. In addition, M468, a triple-negative cell line (estrogen receptor negative, progesterone receptor negative and HER2/neu negative), represents a major subtype of breast cancer more suitable to chemotherapy than hormone therapy due to its hormone independence [64].

### 3.2. Paclitaxel Treatment

The details of paclitaxel treatment are previously described in [22]. In this study we directly measured the changes in calcium release from the ER associated with paclitaxel exposure and dosage using the D1ER cameleon [65]. Briefly, since the actions of paclitaxel on the cells depend on the dose administered and the greatest difference was observed between 10^−6^ and 10^−7^ M, two specific, clinically relevant paclitaxel doses, 2.5 × 10^−6^ M (high dose) and 2.2 × 10^−7^ M (low dose) were used [23,24,66]. Paclitaxel was dissolved in dimethyl sulfoxide (DMSO) solution and then diluted to the desired concentration with medium. DMSO concentrations were kept below 0.5% in all experiments. Both paclitaxel and DMSO were purchased from Sigma (St. Louis, MO, USA). The ER calcium pump inhibitor Thapsigargin (TG), the calcium calibrating agents Ionomycin and EDTA were all obtained from Invitrogen (Carlsbad, CA, USA). In order to study the changes of specific calcium compartments for long-term treatment, a commonly used TG-CaCl_2_ method was used to analyze the ER calcium release and subsequent CCE after long-term (3, 6, 12 h) paclitaxel treatment. While the TG-CaCl_2_ method evaluates ER calcium indirectly through the cytosolic calcium changes induced by the ER release, it can also give a general evaluation of the calcium mobilization among the ER, cytosol, and extracelluar compartments, which was needed for this research. The description of mechanism for this commonly used TG-CaCl_2_ method follows. The addition of Thapsigargin (TG), a highly specific SERCA pump inhibitor, irreversibly blocks the calcium pumping from the cytosol into the ER lumen. TG binds to the functional cavity of SERCA on the ER membrane and locks the pump into a conformation with poor calcium and ATP affinity [67,68]. Although SERCA and the plasma membrane calcium ATPase pump (PMCA) share many structural similarities, TG is ineffective against PMCA. Previous studies have shown that the PMCA pump lacks Glu residue involved in calcium translocation in the SERCA [69]. The structural difference between SERCA and PMCA and specific structural requirement for TG binding may explain the different actions of TG [67–71].

### 3.3. Apoptosis Measurements

Changes in apoptosis levels of cultured cells under different treatments, were measured by double labeling using an Annexin V-Fluorescein isothiocyanate (FITC, green) and Propidium Iodide (PI, red) Apoptosis Kit (Biovision, Mountain View, CA, USA). After trypsinization, the suspended cells were stained with Annexin V-FITC and PI according to the manufacturer’s instructions. Then cells were submitted to an Epics XL-MCL flow cytometer (Beckman Coulter, Miami, FL, USA). Between 5000 and 10,000 cells were analyzed per run. The Annexin V-FITC green signals, localizing to the cell membrane, were proportional to apoptosis events. PI signals, bound to nuclear material, would either be accompanied by green fluorescence signal or be visualized alone, allowing differentiation of early or late apoptotic events. Green signal alone was indicative of early apoptosis, while in combination it signified late apoptosis. PI observed alone indicated a cell necrotic event. The Annexin V-FITC and the PI signals were quantitated by flow cytometry (channels FL1, 526 nm emission = green and FL3, 620 nm emission = red) on a FACSCalibur (Becton Dickenson, Franklin Lakes, NJ, USA).

### 3.4. Cytosolic Calcium Measurements

To detect cytosolic calcium changes, the calcium-specific fluorescent dye Fluo4-AM (Molecular Probes, Eugene, OR, USA), was loaded into M468 cells using a modified procedure adapted from the manufacturer (Molecular Probes, Eugene, OR, USA). The equipment and time-lapsed fluorescence measurement methodology was previously described in [22]. Briefly, The Fluo4-AM (Molecular Probes, Eugene, OR, USA) green fluorescence, ex 488 nm/em 526 nm, was proportional to cytosolic calcium levels. The dynamic changes of Fluo4 intensity over time in cell populations, in response to different agents, were monitored using flow cytometry (FL1 channel) and by deconvolution fluorescence microscopy using Hamamatsu Orca—ER high-speed camera on an Olympus IX-71 inverted microscope (Olympus, Shinjuku, Tokyo, Japan).

### 3.5. Capacitative Calcium Entry Measurements

To quantitate external calcium influx through CCE, coupled with ER calcium depletion, we needed to separate the two possible sources for global cytosolic calcium increase. In order to accomplish this, the TG-CaCl_2_ Method was employed. First, cells were transferred into a calcium free medium to remove the effect of external calcium (Step 1). In the absence of external calcium, Thapsigargin (TG), a highly specific SERCA pump inhibitor, was added to determine ER calcium release [68–71]. The ineffective SERCA pump fails to compensate for passive release of calcium by the ER. Therefore, any measurable cytosolic calcium increase upon TG addition is from the ER store. After cells mobilize calcium efflux through plasma membrane pumps or exchangers to remove the increased cytosolic calcium, the peak cytosolic calcium level would return back to the basal level (Step 2). Then a CaCl_2_ solution was added to measure influx of external calcium. Since the ER was already depleted, the subsequent cytosolic calcium increase was derived from CCE. The final concentration of TG and CaCl_2_ in the medium was 3 μM and 2 mM, respectively. This methodology was performed under normal calcium conditions (Figures 1 and 2), and calcium-free conditions (Figures 5 and 6).

### 3.6. Statistical Analyses

Data from this study were generated from three independent experiments. Apoptosis levels measured were represented as mean ± SD (standard deviation) in the figures. In order to show the calcium changes over time clearly, calcium curves were represented as mean values in the figures, but peak levels were shown as mean ± SD in the additional figures for comparison. Statistical analysis was performed with a two-sided independent Student *t*-test to compare two means, and One-way analysis of variance (ANOVA) to compare more than two means of one variable. If One-way ANOVA demonstrated unequal means, the Tukey’s-honestly significant difference (HSD) test was used to determine which mean was different by conducting multiple pair-wise comparisons. For all analyses, differences with *p* < 0.05 were considered statistically significant and indicated with *, *p* < 0.01 was indicated with **.

### 3.7. Source Code for Paclitaxel Docking Analysis

In order to identify potential targets for paclitaxel binding, a novel docking program named *Artemis* was created. For purposes of proposing alternative mechanisms for paclitaxel regulation of calcium, and based on recent studies, the 20 highest priority molecules were included. The source code is included as Supplementary Material for use in recapitulating the total number of possible docking partners. *Artemis* is a system for docking a single ligand to a library of proteins. *Artemis* requires python (tested with version 2.6, Python Software Foundation, Fredericksburg, VA, USA) and a couple of python libraries to run (*i.e.*, mpi4py and MySQLdb), in addition Autodock Vina (http://autodock.scripps.edu/) needs to be installed. Two libraries, “pdb_subset.py” and “pdb_centermass.py” from the pdb-tools project (https://code.google.com/p/pdb-tools/) were modified and used in *Artemis*. Furthermore, a MySQL database (Oracle Corporation, Redwood City, CA, USA) is required, the structure of which is given in “*Artemis*.sql”. *Artemis* should be deployed in an MPI compatible high performance computing environment for parallel processing of docking jobs.

The database which *Artemis* uses is composed of 9 tables. A brief description of each table follows. The “PDB” Table contains information of a protein in PDB format (*i.e.*, RCSB identifier, title of file, source of file, and organism). Each entry in the “PDB_FILE” Table contains the contents of a PDB file and is associated to an entry in the “PDB” Table. The “CHAINS” Table contains the names of protein chains and is associated to a source PDB in the “PDB” Table. The “CHAINS_FILE” Table contains the subset of a PDB file corresponding to an entry in the “CHAINS” Table. The “LIGANDS” Table contains information regarding a ligand (*i.e.*, name and origin). The “LIGANDS_FILE” Table contains the structure of ligands in PDB format each with a respective entry in the “LIGANDS” Table. The “RESULTS” Table contains the results of a docking job (*i.e.*, associated raw result file, protein chain, ligand, index in result file, energy, root-mean-square deviation lower bound, and root-mean-square deviation upper bound). The “RESULTS_FILE” Table contains the raw output of a docking job. Finally, the “BATCH” Table contains information regarding individual docking jobs (*i.e.*, protein chain, ligand, status of a docking job, and start time of a docking job).

*Artemis* operates as follows: First, the protein library, in PDB format, must be uploaded to the MySQL database. The script “pdb_push.py” is used for this procedure. The script reads in a PDB file, uploads the contents to the database, then using the library “pdb_subset.py” splits the protein into its component chains. Any ligands that are included with the PDB file are discarded. The component chains are then also uploaded to the database. Ligands may also be uploaded using “pdb_push.py”. In order to create a docking job, a SQL script must be written, an example is given in “Batch.sql”. The library “batch.py” contains all the necessary functions for performing a docking job; it is used by “*Artemis*.py”. The library “pdb_centermass.py” computes the center of mass of a protein. The script “*Artemis*.py” is the main application and uses MPI to distribute docking jobs to computational nodes and process them; it also manages the “BATCH” Table by updating the status of docking jobs. A web application, written in PHP, for viewing results is also provided in the scripts “Query.php” and “Result.php”.

An example of a typical docking job is given. First, “*Artemis*.py” is executed on multiple compute nodes. The master node (*i.e.*, the node with MPI rank 0) will retrieve from the database a list of docking jobs from the “BATCH” Table. A docking job consists of a unique database identifier, a ligand, and a protein chain. Each compute node is then given an individual docking job. A compute node will update the status of the docking job in the database as it proceeds. A compute node will retrieve from the database the structure of the protein chain and ligand to be docked. The center of mass of the protein chain is determined; this will be the center of the search space. Autodock Vina is then called to perform the actual docking of the ligand to the protein chain. By default the search space is 30 Angstroms in the *X*, *Y*, and *Z* directions. The results of the docking job are then uploaded to the database. The compute node will then retrieve another docking job and begin the process again. Results can be queried by the user using the provided PHP web application or by querying the database directly using SQL.

## 4. Conclusions

**Figure 7.**
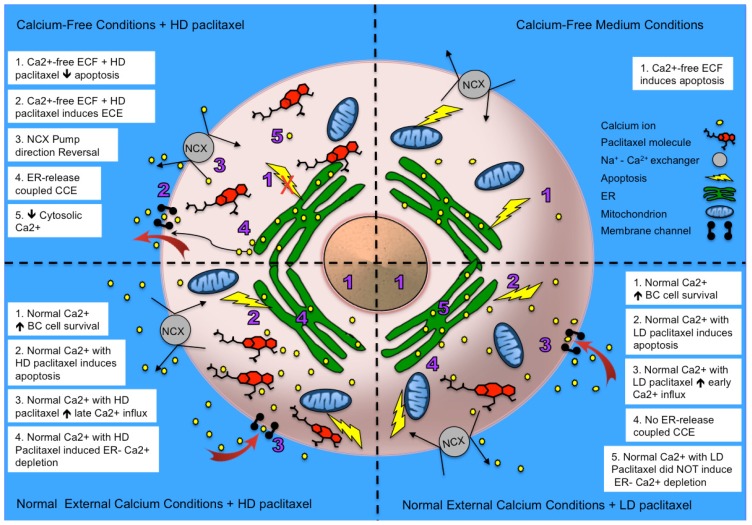
Graphical summary of key results. A schematic of a breast cancer cell from this study is divided into four quadrants. Each quadrant describes the external calcium conditions as well as paclitaxel treatment. Numbers for each key point are correspondingly depicted within the cell quadrant. HD = high dose paclitaxel, LD = low dose paclitaxel, NCX = sodium calcium exchange pump.

Our results showed that paclitaxel can enhance the cell’s influx of external calcium, and the contribution of enhanced calcium influx and underlying mechanisms depend on dosage of paclitaxel. Our findings are summarized graphically in Figure 7. In summary, we found that normal external calcium conditions are required to maintain cell survival in breast cancer cells, and long-term absence of external calcium alone can result in apoptosis. While high dose paclitaxel-enhanced CCE depends on the involvement of ER calcium release, low dose paclitaxel did not involve the ER calcium-regulated pathway. We also observed an antagonistic relationship between the two apoptosis-generating factors (e.g., paclitaxel treatment and external calcium removal). Although either alone can induce apoptosis, no additive or synergistic effects on apoptosis were observed when cells were treated with paclitaxel in the absence of extracellular calcium. In fact, the results indicated that an antagonizing effect exists between the two apoptosis-generating factors. Since high dose paclitaxel and external calcium removal oppose each other in the induction of apoptosis, we further dissected the relationship between them by testing whether external calcium removal directly mediated a high dose paclitaxel-induced cytosolic calcium change, which is the intermediate step in paclitaxel-induced apoptosis. Indeed, high dose paclitaxel, in the absence of external calcium, induces an Enhanced Calcium Efflux (ECE), which may be enabled through ER-facilitated efflux, NCX reversal [72], or a combination, to refill the immediate extracellular environment. This efflux mechanism for high dose paclitaxel may explain its different effects on calcium homeostasis, and the different stimulatory and inhibitory effects on subsequent apoptosis. In summary, in control medium with the normal external calcium conditions, paclitaxel caused an increase in external calcium influx to the cytosol, which promoted subsequent apoptosis. However, in the absence of external calcium, paclitaxel could not mobilize the external calcium source, and the subsequent calcium-dependent apoptosis was inhibited. Although the results cannot be directly generalized to clinical situations, the external calcium level in the medium is representative of the plasma calcium level, and suggests that the patient’s plasma calcium level may need to be considered when evaluating the efficacy of chemotherapy, especially in clinical situations such as hypocalcaemia. Further studies in translational and clinical research are needed in this field.

## Figures and Tables

**Table 1.** Top twenty docking results identified using the paclitaxel ligand docking program *Artemis*.

Priority Docking Number	RCSB_ID	Energy (kcal/mol)	Species	Protein
1	2ekg	−10.5	*Thermus thermophilus*	flavoenzyme proline dehydrogenase
2	2h7m	−10.4	*Mycobacterium tuberculosis*	enoyl reductase
3	2i6x	−10.3	*Porphyromonas gingivalis*	hydrolase
4	3ek2	−10.3	*Burkholderia pseudomallei*	eonyl reductase
5	3g7w	−9.9	*Homo sapiens*	Islet Amyloid Polypeptide
6	2xn5	−9.9	*Homo sapiens*	Thyroxine-Binding Globulin
7	3i6i	−9.9	*Vitis vinifera*	leucoanthocyanidin reductase
8	1hvb	−9.7	*Streptomyces*	DD-Peptidase
9	3i6o	−9.6	*HIV-1*	HIV-1 protease
10	1hv8	−9.6	*Hyperthermophile* *Methanococcus Jannaschii*	Dead Box Protein
11	2prb	−9.6	*Salmonella typhimurium*	coenzyme A
12	1ekk	−9.6	*Bacillus subtilis*	Hydroxyethylthiazole Kinase
13	1xpj	−9.5	*Vibrio cholerae*	MCSG Target APC26283
14	3h2h	−9.4	*Xanthomonas oryzae*	esterase LipA
15	1xfs	−9.6	*Nitrosomonas europaea*	Protein NE0264
16	1set	−9.5	*Thermus thermophilus*	Seryl-TRNA Synthase
17	2h7x	−9.3	*Streptomyces venezuelae*	Pikromycin Thioesterase
18	3hv5	−9.3	*Homo sapiens*	p38 MAP Kinase
19	2xff	−9.3	*Hordeum vulgare*	Beta-Amylase
20	1yp3	−9.7	*Solanum tuberosum*	ADP-glucose pyrophosphorylase
